# Nanoporosity of Carbon–Sulfur Nanocomposites toward the Lithium–Sulfur Battery Electrochemistry

**DOI:** 10.3390/nano11061518

**Published:** 2021-06-08

**Authors:** Chien-Hsun Yu, Yin-Ju Yen, Sheng-Heng Chung

**Affiliations:** 1 Department of Materials Science and Engineering, National Cheng Kung University, No.1, University Road, Tainan City 701, Taiwan; hankchienhsun@gmail.com (C.-H.Y.); n56094261@gs.ncku.edu.tw (Y.-J.Y.); 2 Hierarchical Green-Energy Materials Research Center, National Cheng Kung University, No.1, University Road, Tainan City 701, Taiwan

**Keywords:** electrochemistry, porosity, lithium–sulfur battery, porous carbon, polysulfide, high loading

## Abstract

An ideal high-loading carbon–sulfur nanocomposite would enable high-energy-density lithium–sulfur batteries to show high electrochemical utilization, stability, and rate capability. Therefore, in this paper, we investigate the effects of the nanoporosity of various porous conductive carbon substrates (e.g., nonporous, microporous, micro/mesoporous, and macroporous carbons) on the electrochemical characteristics and cell performances of the resulting high-loading carbon–sulfur composite cathodes. The comparison analysis of this work demonstrates the importance of having high microporosity in the sulfur cathode substrate. The high-loading microporous carbon–sulfur cathode attains a high sulfur loading of 4 mg cm^−2^ and sulfur content of 80 wt% at a low electrolyte-to-sulfur ratio of 10 µL mg^−1^. The lithium–sulfur cell with the microporous carbon–sulfur cathode demonstrates excellent electrochemical performances, attaining a high discharge capacity approaching 1100 mA∙h g^−1^, a high-capacity retention of 75% after 100 cycles, and superior high-rate capability of C/20–C/3 with excellent reversibility.

## 1. Introduction

Lithium-ion (Li-ion) battery technology has dominated the rechargeable energy-storage market since the 1990s. With the continuous optimization of battery science, various Li-ion battery cathodes have almost attained their theoretical capacities of 150–300 mA∙h g^−1^ and their promised reversible cyclability [1,2,3,4,5]. To adapt the Li-ion technology for high-energy-density applications, it is critical to develop high-capacity cathodes with highly reversible battery chemistry beyond those of current Li-ion batteries. The lithium–sulfur (Li–S) battery cathode has drawn increasing attention due to its superior theoretical capacity of 1675 mA∙h g^−1^ and high reversible cyclability, resulting in a high energy density of 2600 W∙h kg^−1^. Moreover, the sulfur cathode has the advantages of being naturally abundant, inexpensive, and eco-friendly [1,4,5,6].

However, the commercialization of Li–S batteries is hampered by several intrinsic material issues. Solid-state sulfur and its end-discharge products (Li_2_S/Li_2_S_2_) are insulating active materials, meaning that a sulfur cathode suffers from low electrochemical utilization, high cycle irreversibility, and poor rate capability [7,8,9,10]. In the intermediate charge/discharge states, the liquid-state lithium polysulfides generated (Li_2_S_4-8_) are soluble in the liquid electrolyte. The dissolved polysulfides that have strong mobility and activity would diffuse out of the cathode, relocate in the cell, and react with the lithium metal anode and electrolyte. The irreversible polysulfide diffusion and the resulting consumption of the electrodes and electrolyte will inevitably lead to poor cycle life and low discharge/charge efficiency [6,7,8,9,10]. These material issues also generate additional extrinsic fabrication problems, such as a low sulfur loading of less than 2 mg cm^−2^ and an insufficient sulfur content of less than 60 wt% in the cathode. The low amount of sulfur in the cathode hampers the design of a high-energy-density Li–S cathode [3,4,11]. To address these issues, great effort has been devoted to the encapsulation of the insulating sulfur in various porous conductive carbon substrates to yield a carbon–sulfur (C–S) nanocomposite, which improves the charge-transfer capability and absorbs the dissolved polysulfides [12,13,14,15,16,17,18]. Although different polar materials are subsequently reported to have a strong chemical polysulfide-trapping capability, these materials must be either mixed with, generated on, or made to decorate a porous conductive carbon substrate to obtain their promising performance in high-capacity retention [19,20,21,22,23,24,25]. This means that a C–S nanocomposite study might be the foundation for developing a Li–S battery cathode that enables the use of a large amount of insulating active material to attain high-performance electrochemical characteristics [12,13,14,15,16,17,18,19,20].

Accordingly, in this paper, we examine the effects of the nanoporosity of various porous conductive carbon substrates on the electrochemical characteristics and cell performances of the resulting C–S nanocomposites. We controlled the sulfur content in the synthesized C–S nanocomposites using the sulfur-melting method to obtain a nearly constant sulfur content as high as 80 wt%, and we used the drop-casting method to attain a fixed high sulfur loading of 4 mg cm^−2^ with no additional conductive additives or binders. Thus, the carbon substrates applied in various C–S nanocomposites could be studied to determine the effect of their nanoporosity on the Li–S electrochemistry and the feasibility of their use in a high-loading sulfur cathode with high energy density. Four commonly used carbon substrates were analyzed and categorized as either nonporous, microporous, micro/mesoporous, or macroporous. Among the resulting C–S nanocomposites, the microporous C–S nanocomposite displays strong retention of the active material, which attains a high charge-storage capacity of 1084 mA∙h g^−1^, a high reversible capacity of 817 mA∙h g^−1^ after 100 cycles, and a high rate capability from C/20 to C/3 rates, exhibiting high reversibility and long-term cyclability at a low electrolyte-to-sulfur ratio of 10 µL mg^−1^. As a result of these enhanced electrochemical characteristics, the high-loading microporous C–S composite cathode demonstrates a high areal capacity of 4.3 mA∙h cm^−2^ and a high energy density of 9.2 mW∙h cm^−2^. Therefore, we suggest the adoption of a carbon substrate featuring a high micropore area and volume in the development of a C–S composite cathode with superior electrochemical characteristics and a high amount of sulfur.

## 2. Experimental Procedure

### 2.1. Carbon–Sulfur (C–S) Cathode Preparation

The carbon substrates selected for this study include four commercial conductive carbon blacks, Super P (Super P Conductive, Alfa Aesar, Ward Hill, MA, USA), Black Pearls (BP2000, CABOT, Boston, MA, USA), Ketjenblack (EC-600JD, AkzoNobel, Amsterdam, The Netherlands), and Vulcan Black (VXC72, CABOT, Boston, MA, USA). Based on their nitrogen adsorption–desorption behavior and porosity analysis results, they were categorized as nonporous, microporous, micro/mesoporous, or macroporous carbons, respectively. The various carbon blacks were then ground and mixed with sulfur (99.5%, Acros Organics, Fair Lawn, NJ, USA) in a fixed weight ratio of 20:80 for 30 min at room temperature (25 °C). The C–S mixtures were then transferred to a preheated electric oven and isothermally heated at 155 °C for 6 h in an argon-filled glass vessel, resulting in the formation of gray-black C–S nanocomposites with a constant sulfur content of ~80 wt% that was analyzed by thermogravimetric and elemental analysis.

### 2.2. Materials Characterization

The porosities and surface science of the carbon blacks were analyzed using a gas sorption analyzer (Autosorb iQ MP/MP, Anton Paar, Graz, Austria) from 0.00001 to 1.0 P/P_0_ at 77 K. The specific surface area (SSA), total pore volume (TPV), and average pore size were calculated based using the Brunauer–Emmett–Teller (BET) theory and T-plot analysis. The pore size distribution (PSD) was simulated by Horvath–Kawazoe (HK) theory, density functional theory (DFT), and Barrett–Joyner–Halenda (BJH) theory. The morphology, microstructure, and elemental distribution of the carbon blacks and the C–S nanocomposites were characterized using a field-emission scanning electron microscope (FE-SEM, SU-5000, HITACHI, Tokyo, Japan) equipped with an energy dispersive X-ray spectrometer (EDS). The structure, elemental composition, and sulfur content of the C–S nanocomposites were analyzed using an X-ray diffractometer (XRD, D8 DISCOVER, Bruker, Billerica, MA, USA), an elemental analyzer (EA, UNICUBE, Elementar, Langenselbold, Germany), and a thermogravimetric analyzer (TGA, 7, Perkin-Elmer, Waltham, MA, USA), respectively.

### 2.3. Electrochemical Characterization

The C–S nanocomposite was first mixed with an electrolyte solution to form a paste. The mixture paste of electrolyte and active material was then drop-casted onto a carbon current collector (Nanolab) to produce a C–S composite cathode with no additional conductive carbons or binders. The resulting high-loading sulfur cathode had a fixed high sulfur content of 80 wt% and a constant high sulfur loading of 4 mg cm^−2^. The electrolyte solution contained 1.85 M lithium bis(trifluoromethanesulfonyl)imide (LiTFSI, 98%, Acros Organics, Fair Lawn, NJ, USA) and 0.1 M lithium nitrate (99+%, Acros Organics, Fair Lawn, NJ, USA) in a mixed 1,3-dioxolane (99+%, Acros Organics, Fair Lawn, NJ, USA) and 1,2-dimethoxyethane (99.8%, Acros Organics, Fair Lawn, NJ, USA) solvent. Lithium–sulfur cells were assembled with the C–S composite cathode, a polypropylene membrane (Celgard, Charlotte, NC, USA), and a metallic lithium counter electrode (99.9%, trace metal basis, Sigma-Aldrich) with a low electrolyte-to-sulfur ratio of 10 µL mg^−1^ [3,4] in an argon-filled glove box with low levels of H_2_O and O_2_ (H_2_O < 0.1 ppm, O_2_ < 0.1 ppm). A reference sulfur cathode was prepared by the conventional method with 75 wt% sulfur, 19 wt% Super P (Super P Conductive), and 6 wt% PvDF binder. The reference cathode had the same sulfur loading of 4 mg cm^−2^ and similar S:C ratio of 4:1 as compared to those of the C-S composite cathode. The resulting cells were analyzed using a programmable battery cycler (BCS-800 series, Biologic, Seyssinet-Pariset, France) within a voltage window of 1.8–2.8 V at the C/10 rate to test their long-term cycling performance and at various cycling rates from the slow C/20 rate to the fast C/3 rate to determine their rate capabilities. The rate-performance testing subsequently changed to C/20 and C/10 rates for an additional 100 cycles for examining the electrochemical reversibility and stability of the C–S composite cathodes. The electrochemical impedance analysis was investigated by a potentiostat (SP150, Biologic, Seyssinet-Pariset, France) with a frequency range from 1 MHz to 0.1 Hz and an amplitude of 5 mV. The electrochemical characteristics and cell performance were evaluated based on the cycling performance, the rate capability, the discharge/charge voltage profiles, and the polarization. The current density (1 C = 1675 mA g^−1^) and the specific capacity were based on the sulfur loading in the cathode.

## 3. Results and Discussion

### 3.1. Material Characteristics of Various Carbon Substrates and C–S Nanocomposites

**Figure 1:**
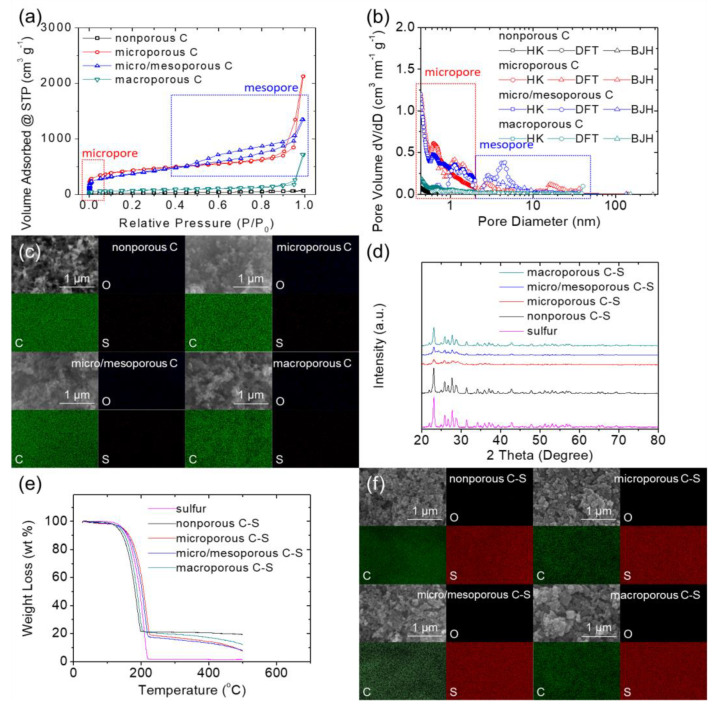
Material characteristics: (**a**) nitrogen adsorption–desorption isotherms (ISOs), (**b**) pore size distributions (PSDs), and (**c**) scanning electron microscopy and energy dispersive X-ray spectrometry (SEM/EDS) of various carbons. (**d**) X-ray diffraction (XRD), (**e**) thermogravimetric analysis (TGA), and (**f**) SEM/EDS of various carbon–sulfur (C–S) nanocomposites.

Figure 1 shows a summary of the material characteristics of various carbon blacks and their corresponding C–S nanocomposites. Figure 1a,b show the nitrogen adsorption–desorption isotherms (ISOs) and pore size distribution (PSD) analysis, in which the nonporous carbon (black line) showed weak adsorption and a limited distribution of nanopores. The microporous carbon (red line) exhibited an IUPAC type-I ISO at a low P/P_0_ ratio of less than 0.1, indicating strong micropore adsorption behavior [26]. Its PSD analysis confirmed the abundance of micropores. The micro/mesoporous carbon (blue line) exhibited a distinguishable type-IV mesopore hysteresis loop at 0.4–0.9 P/P_0_ ratios [26], corresponding to the mesopore peaks at 3–4 nm detected in the PSD analysis. The macroporous carbon (green line) displayed no microporosity or mesoporosity, but a normal type-II ISO with macropores was evident in the high P/P_0_ region [26]. Based on the data obtained, we determined the specific surface area (SSA) and total pore volume (TPV) of the carbon blacks. The nonporous carbon had the lowest SSA and TPV values of 79 m^2^ g^−1^ and 0.10 cm^3^ g^−1^, respectively, and no micropores. In sharp contrast, the microporous carbon exhibited the highest SSA and TPV values of 1435 m^2^ g^−1^ and 3.30 cm^3^ g^−1^, respectively, and more than half of its high SSA was a result of its micropores. Unlike the microporous carbon, although the micro/mesoporous carbon also displayed high SSA and TPV values of 1301 m^2^ g^−1^ and 2.09 cm^3^ g^−1^, respectively, mesopores dominated. The macroporous carbon has SSA and TPV values of 239 m^2^ g^−1^ and 1.11 cm^3^ g^−1^, respectively, with a limited distribution of micropores and mesopores. The ISO, PSD, and porosity results confirmed the unique nanoporosities of each carbon black. These analyzed morphological properties of the four carbon blacks are summarized in Table S1. Figure 1c shows a summary of the microstructure and elemental mapping results of the carbon substrates, indicating that the carbon blacks are spherical carbons with similar nanosized clusters.

Figure 1d shows a comparison of the X-ray diffraction (XRD) patterns of various C–S nanocomposites and sulfur. The nonporous C–S nanocomposite showed strong sulfur patterns, implying that the melting sulfur might cover and crystallize outside the nonporous carbon black. With abundant nanopores and a high TPV, the sulfur-based nanocomposites with microporous and micro/mesoporous carbons featured an obvious decrease in sulfur peak intensity. The difference in these XRD patterns indicates that porous carbon with a high nanoporosity would allow the infusion and uniform dispersion of sulfur in its nanoporous framework, resulting in a weak sulfur XRD peak [16,19,20]. The XRD pattern of the macroporous C–S nanocomposite was consistent with these phenomena; it exhibited detectable sulfur peaks with an intensity between that of nonporous and highly porous carbon, as the limited TPV could not accommodate all the sulfur. The excess sulfur thus recrystallized outside the porous framework on the particle surface and was, therefore, observable in the XRD patterns [19,20]. Figure 1e shows the thermogravimetric analysis (TGA) data, affirming that all the C–S nanocomposites have a similar sulfur content of 80 ± 1 wt% (Table S2). The highly porous carbons featured strong sulfur accommodation at a higher decomposition temperature and a somewhat high sulfur content in their porous spaces [12,20]. The elemental analysis reconfirmed the relatively high sulfur content in the microporous and micro/mesoporous C–S nanocomposites (Table S2). Figure 1f shows the morphological changes in the carbon blacks after sulfur-melting treatment. As they were hosting sulfur, the C–S nanocomposites exhibited slightly higher granularities and particle sizes than the carbon blacks. The corresponding elemental mapping results also indicated the strong homogeneous sulfur signal of the nanocomposites.

### 3.2. Electrochemical Analysis and Performance of Various C–S Nanocomposites

**Figure 2:**
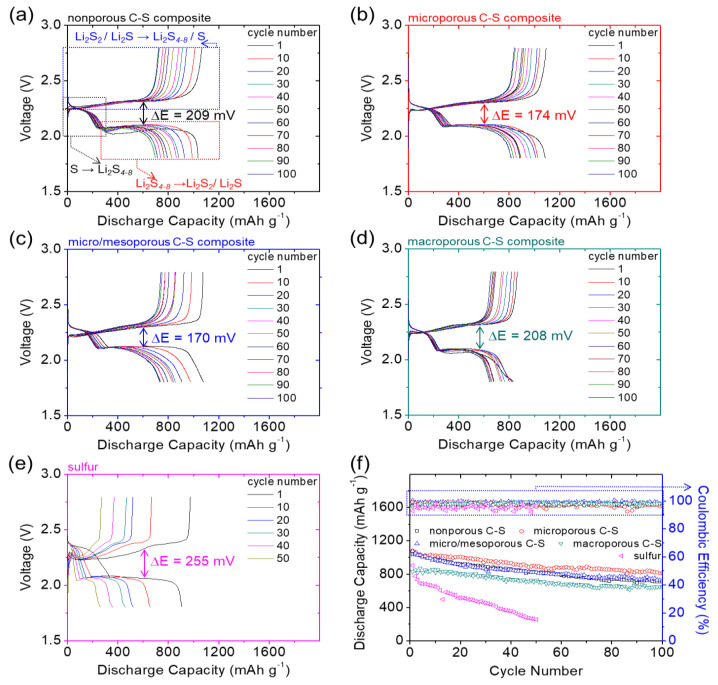
Electrochemical analysis of various carbon–sulfur (C–S) nanocomposites: voltage profiles of (**a**) nonporous C–S, (**b**) microporous C–S, (**c**) micro/mesoporous C–S, (**d**) macroporous C–S, and (**e**) sulfur; (**f**) cyclability.

Figure 2 shows a summary of the electrochemical characteristics of various C–S nanocomposites. Figure 2a–d depict the voltage profiles of the nanocomposites. The redox process involved the reduction of sulfur to polysulfides at ~2.3 V (marked with a black box) and the subsequent conversion from polysulfides to sulfides at ~2.1 V (marked with a red box) during discharge [9,21,22,23]. The initial discharge capacity values of the nonporous, microporous, micro/mesoporous, and macroporous C–S nanocomposites attained 1036, 1084, 1077, and 831 mA∙h g^−1^, respectively, at the C/10 rate. The trend of electrochemical utilization of the C–S nanocomposites agrees with the EIS data, which displays a low cell impedance of the nonporous, microporous, and micro/mesoporous C–S nanocomposites, while a slightly high impedance in the macroporous C–S nanocomposites (Figure S1). While charging, the reversible oxidizations from sulfides to polysulfides and sulfur were characterized by a continuous charge curve at ~2.3 V (marked with a blue box) [9,23]. All the C–S nanocomposites exhibited overlapping discharge and charge curves without a significant decline in the charge-storage capacity values in successive cycles. This demonstrated their enhanced electrochemical utilization and stability [8,9,11,23]. Thus, although a high amount of insulating sulfur (i.e., 4 mg cm^−2^ and 80 wt%) was present in the composite cathode, the C–S nanocomposites maintained low polarization. The microporous and micro/mesoporous C–S nanocomposites exhibited the lowest polarization of 0.17 V, which was lower than those of the nonporous and macroporous C–S nanocomposites (0.21 V). Seemingly, the encapsulation of insulating sulfur within a highly porous conductive carbon substrate accelerates the charge transfer and improves the reaction kinetics [8,9,10,11,16]. Moreover, microporous carbon, which features abundant micropores, a high surface area, and a large pore volume, enables the microporous C–S nanocomposite to attain the highest electrochemical utilization and stability [16,19,20], resulting in the highest reversible capacity and most stable capacity retention (Figure 2b). As a reference, Figure 2e shows the typical two-step redox reaction of a reference sulfur cathode, suffering high and increasing polarization from 0.26 V and rapid capacity loss.

Figure 2f shows the cyclability of the C–S nanocomposites at the C/10 rate. After 100 cycles, the reversible capacity and capacity retention values (in parentheses) of the nonporous, microporous, micro/mesoporous, and macroporous C–S nanocomposites approached 714 (69%), 817 (75%), 732 (68%), and 645 mA∙h g^−1^ (78%), respectively. These cyclability data correspond well with the results of the material characterization and electrochemical analysis, confirming the superior electrochemical utilization and stability of the microporous C–S cathode [20,23]. The micro/mesoporous C–S cathode initially exhibited high electrochemical utilization, but the relatively low SSA/TPV values and the limited microporosity of the micro/mesoporous carbon may have weakened its polysulfide-trapping capability [12,19,20]. The same issues arose in the cases of the nonporous and macroporous C–S nanocomposites, experiencing relatively fast capacity fade and low reversible electrochemical utilization. As a reference, with the same high amount of sulfur, the reference sulfur cathode cannot normally cycle for 50 cycles or more, which shows a continuous capacity drop and unstable Coulombic efficiency. As a result, in Figure 2, we show the enhanced Li–S cathode electrochemical performance obtained by various C–S nanocomposites. Among these nanocomposites, microporous carbon featured a high SSA, large TPV, and strong microporosity, exhibiting high sulfur-loading capability and excellent electrochemical utilization and stability. Therefore, the microporous C–S nanocomposite attained a high areal capacity of 4.3 mA∙h cm^−2^ and energy density of 9.2 mW∙h cm^−2^, surpassing the current criteria for state-of-the-art Li–S cells [1,2,3,4,5,8,11,12,13,14,15,16]. 

### 3.3. Rate-Capability Analysis and Performance of Various C–S Nanocomposites

**Figure 3:**
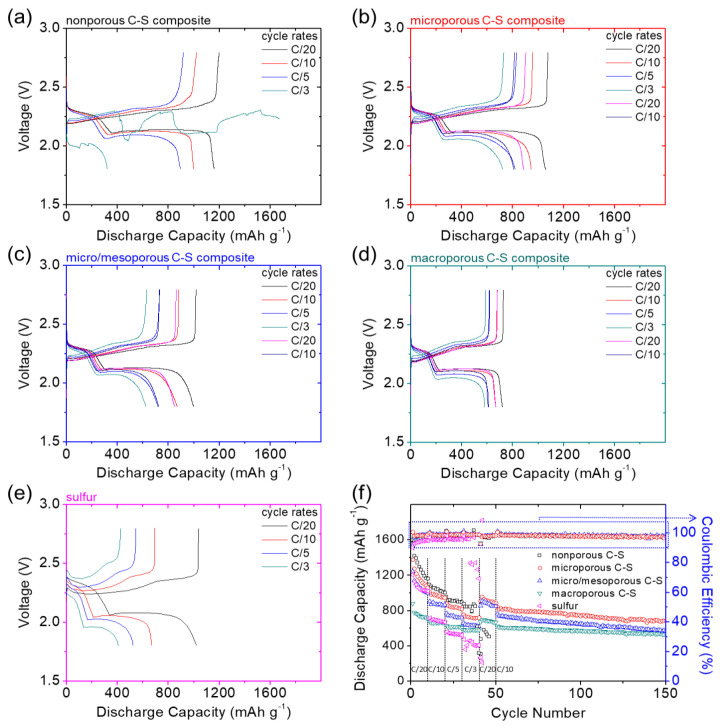
Rate-capability analysis of various carbon–sulfur (C–S) nanocomposites: voltage profiles of (**a**) nonporous C–S, (**b**) microporous C–S, (**c**) micro/mesoporous C–S, (**d**) macroporous C–S, and (**e**) sulfur; (**f**) rate capability.

Figure 3 summarizes the rate capability and subsequent cycle stability of the C–S nanocomposites. Figure 3a–d show the voltage profiles of the nanocomposites at C/20, C/10, C/5, C/3, C/20, and C/10 rates. At the slow C/20 rate, the nonporous, microporous, and micro/mesoporous C–S nanocomposites attained excellent sulfur utilization and high reversible capacity values of 1160, 1059, and 998 mA∙h g^−1^, respectively, at the tenth cycle. However, the macroporous C–S nanocomposite had a relatively low discharge capacity of 718 mA∙h g^−1^. As the cycling rate increases to the fast C/3 rate, nonporous carbon cannot retain normal cyclability and loses its high-rate capability due to the formation of insulating solid-state active materials on its surface (Figure 3a). The microporous and micro/mesoporous carbons that encapsulate the insulating solid-state sulfur and sulfides, and absorb dissolved polysulfides within their nanoporous spaces [12,19,20,21,22], enable the high-loading cathode to attain an enhanced high-rate capability and stability (Figure 3b,c). The macroporous C–S nanocomposite experiences low reaction kinetics and deteriorated polarization with increases in the cycling rates (Figure 3d).

After changing the cycling rate back to the slow C/20 rate, the microporous, micro/mesoporous, and macroporous C–S nanocomposites showed reversible capacity values and corresponding reversibility rates (in parentheses) of 887 (84%), 849 (85%), and 667 mA∙h g^−1^ (93%), respectively, demonstrating the excellent high-rate capability of the microporous C–S nanocomposite featuring high electrochemical utilization and reversibility. As a reference, the sulfur cathode experienced high polarization and a low reaction capability, which were significantly exacerbated with increases in the cycling rates as the cell fails (Figure 3e). As a result, the rate-dependent voltage profiles indicated that microporous carbon boosted the reaction kinetics and electrochemical reversibility of the C–S nanocomposite, whereas micro/mesoporous carbon had a relatively low effect on the performance improvement. In contrast, nonporous carbons might not be able to encapsulate the active material and macroporous carbons would unleash dissolved polysulfides during cycling, resulting in poor rate capability and low cycle reversibility, respectively [11,16,17,18,19,20,21,22]. 

Figure 3f summarizes the rate capability at C/20–C/3 and the subsequent cycle stability at C/20 and C/10 rates for another 100 cycles. At the slow C/20 rate, the nonporous C–S nanocomposite had a high initial discharge capacity of 1593 mA∙h g^−1^. However, the cell encountered a fast capacity decrease in the initial ten cycles and a poor rate capability at the C/3 rate because the nonporous carbon could not achieve a strong polysulfide-trapping capability. In contrast, porous carbons with a higher SSA, larger TPV, and stronger microporosity enabled high-loading sulfur cathodes to attain higher electrochemical utilization, reversibility, and stability at different rates. Among these porous carbons, the microporous C–S nanocomposite attained high reversible capacity and stability at various rates and had the highest electrochemical utilization and cyclability during the subsequent 100 cycles at the C/10 rate. Therefore, from Figure 3, we can conclude that the microporous C–S nanocomposite features excellent rate performance due to its strong encapsulation of insulating sulfur within the conductive microporous carbon framework, facilitating a fast charge transfer and high polysulfide-trapping capability [14,15,16,20,21,22].

### 3.4. Performance Comparison of Various C–S Nanocomposites

**Figure 4:**
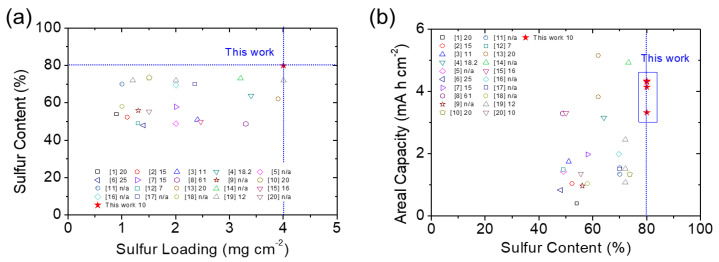
Comparative analysis of the battery performances and electrochemical characteristics of the C–S composite cathode with reported Li–S research using different C–S composites: (**a**) sulfur loading, sulfur content, and electrolyte-to-sulfur ratio (the electrolyte-to-sulfur ratio with a unit as µL mg^−1^ is provided after the number of citations; see supporting information); (**b**) sulfur content, areal capacity, and electrolyte-to-sulfur ratio.

In order to support the abovementioned conclusions, we summarize the cycling performance at the constant C/10 rate and the rate capability from C/20 to C/3 of the four C–S nanocomposites (Table S3 and Figure S2). The comparison demonstrated that the microporous C–S nanocomposite featured the best electrochemical utilization and efficiency; alongside this, it showed excellent reaction kinetics and stability. The excellent electrochemical stability and efficiency of the microporous carbon resulted from its strong microporosity that trapped the active material in its large porous space and, therefore, hosted the sulfur in a conductive matrix [14,15,16,19,20,21,22]. However, all C–S nanocomposites displayed significant improvement in the overall Li–S cell performance in comparison with those obtained by the reference sulfur cathode prepared by the conventional method. In addition to making a comparison within our samples, Figure 4 shows the comparison of our C–S nanocomposites with the reported C–S composite cathodes in the literature in terms of the capability to host a high amount of sulfur and the electrochemical performance at a low electrolyte-to-sulfur ratio. The data comparison demonstrates that the use of microporous C–S nanocomposite by drop-casting the mixture of active material and electrolyte enables the high-loading sulfur cathode (Figure 4a) to attain a high areal capacity at the lean electrolyte condition (Figure 4b). The detailed performance data are summarized in Table S4 as a reference.

## 4. Conclusions

In summary, we systemically investigated the effects of the nanoporosity of various selected carbon blacks on the electrochemical characteristics and battery performance of Li–S cells with a high-loading sulfur cathode that has 4 mg cm^−2^ sulfur loading and 80 wt% sulfur content at a low electrolyte-to-sulfur ratio of 10 µL mg^−1^. Four distinct carbon blacks were characterized and used to produce nonporous, microporous, micro/mesoporous, and macroporous C–S composite cathodes by the sulfur-melting method for the nanocomposite synthesis and the drop-casting method of the mixture of electrolyte and active material on the electrode substrate to form the cathode. Of the different carbon blacks, the conductive microporous carbon that featured high SSA/TPV and abundant micropores enabled the high-loading sulfur cathode to attain superior electrochemical utilization, demonstrating a high areal capacity of 4.3 mA∙h cm^−2^ and a high energy density of 9.2 mW∙h cm^−2^. The microporous C–S nanocomposite exhibited excellent cycle stability during long-term cyclability (75% capacity retention after 100 cycles) and rate-performance testing (from C/20 to C/3 rates) with excellent cyclability for another 100 cycles.

## Data Availability

The data presented in this study are available in this study and the corresponding supplementary material.

## Supplementary Materials

The following are available online at https://www.mdpi.com/article/10.3390/nano11061518/s1, Table S1: Morphological properties of carbon blacks; Table S2: Material analysis of C–S nanocomposites; Table S3: Electrochemical performance of C–S nanocomposites; Table S4: Comparative analysis of the battery performances and electrochemical characteristics of the C–S composite cathodes in the Li–S research; Figure S1: Electrochemical impedance analysis of C–S nanocomposites; Figure S2: The effect of nanoporosity of C–S nanocomposites toward their corresponding electrochemical performance.
