# Supplementary material for: Nanoporosity of Carbon–Sulfur Nanocomposites toward the Lithium–Sulfur Battery Electrochemistry
Source: Nanomaterials (Basel). 2021 Jun 8;11(6):1518. doi: 10.3390/nano11061518 (PMC8228317; doi:10.3390/nano11061518)
Supplement: Supplementary file 1 [file nanomaterials-11-01518-s001.zip › nanomaterials-1239292-supplementary.pdf]

nanomaterials

*Supporting Information*

# Nanoporosity of Carbon–Sulfur Nanocomposites toward the Lithium–Sulfur Battery Electrochemistry

**Chien-Hsun Yu [1], Yin-Ju Yen [1] and Sheng-Heng Chung [1,2,*]**

[1] Department of Materials Science and Engineering, National Cheng Kung University, No.1, University Road, Tainan City 701, Taiwan; hankchienhsun@gmail.com (C.-H. Y.); n56094261@gs.ncku.edu.tw (Y.-J. Y.)
[2] Hierarchical Green-Energy Materials Research Center, National Cheng Kung University, No.1, University Road, Tainan City 701, Taiwan
* Correspondence: SHChung@gs.ncku.edu.tw

**Table S1.** Morphological properties of carbon blacks.

| | Nonporous carbon | Microporous carbon | Micro/mesoporous carbon | Macroporous carbon |
|---|---|---|---|---|
| Specific surface area ($m^2$ $g^{-1}$) | 79 | 1435 | 1301 | 239 |
| Particle size (nm) | 40 | 35 | 35 | 50 |
| Total pore volume ($cm^3$ $g^{-1}$) | 0.10 | 3.30 | 2.09 | 1.11 |
| Average pore diameter (nm) | 5.09 | 9.20 | 6.42 | 18.52 |
| Micropore surface area ($m^2$ $g^{-1}$) | 0.00 | 760 | 77 | 63 |
| Micropore volume ($cm^3$ $g^{-1}$) | 0.00 | 0.38 | 0.03 | 0.03 |

**Table S2.** Material analysis of C–S nanocomposites.

| | Nonporous carbon | Microporous carbon | Micro/mesoporous carbon | Macroporous carbon |
|---|---|---|---|---|
| Weight loss (wt%) by TGA | 78.7 | 80.7 | 81.3 | 78.7 |
| Sulfur (wt%) by EA | 79.2 | 80.2 | 80.0 | 79.9 |
| Carbon (wt%) by EA | 19.2 | 18.0 | 18.1 | 18.6 |
| Nitrogen / oxygen / hydrogen (wt%) by EA | 1.6 | 1.8 | 1.9 | 1.5 |

**Table S3.** Electrochemical performance of C–S nanocomposites.

| | Nonporous carbon | Microporous carbon | Micro/mesoporous carbon | Macroporous carbon |
|---|---|---|---|---|
| Cyclability test C/10 the 1st discharge capacity | 1036 | 1084 | 1078 | 831 |
| Rate capability test C/20 the 10th discharge capacity | 1160 | 1059 | 998 | 718 |
| Rate capability test C/10 the 10th discharge capacity | 1001 | 944 | 868 | 667 |
| Rate capability test C/5 the 10th discharge capacity | 894 | 819 | 718 | 612 |

| | | | | |
|---|---|---|---|---|
| Rate capability test C/3 the 10th discharge capacity | 319 | 720 | 622 | 581 |
| Rate capability test C/20 the 10th discharge capacity | un-cyclable | 887 | 849 | 667 |

**Table S4.** Comparative analysis of the battery performances and electrochemical characteristics of the C–S composite cathodes in the Li–S research.

| Sulfur loading (mg cm$^{-2}$) | Sulfur content (wt%) | Electrolyte-to-sulfur ratio (μL mg$^{-1}$) | Cycling rate | Cycle life | Highest capacity (mA h g$^{-1}$) | Areal capacity (mA h cm$^{-2}$) | Reference |
|---|---|---|---|---|---|---|---|
| 0.9 | 54 | 20 | 5C | 400 | 450 | 0.41 | [1] |
| 1.1 | 52.2 | 15 | C/2 | 200 | 955 | 1.05 | [2] |
| 2.4 | 51 | 11 | 1C | 300 | 732 | 1.76 | [3] |
| 3.4 | 64 | 18.2 | 2C | 500 | 932 | 3.17 | [4] |
| 2 | 49 | n/a | C/2 | 500 | 715 | 1.43 | [5] |
| 1.4 | 48 | 25 | 1.5C | 400 | 600 | 0.84 | [6] |
| 2 | 58 | 15 | C/2 | 200 | 992 | 1.98 | [7] |
| 3.3 | 48.8 | 61 | C/2 | 200 | 1000 | 3.30 | [8] |
| 1.3 | 56 | n/a | C/2 | 300 | 750 | 0.98 | [9] |
| 1.5 | 73.6 | 20 | C/5 | 100 | 900 | 1.35 | [10] |
| 1 | 70 | n/a | C/5 | 100 | 1,343 | 1.34 | [11] |
| 1 | 70 | n/a | C/2 | 100 | 1,544 | 1.54 | [11] |
| 1.3 | 49 | 7 | C/10 | 100 | 1,150 | 1.50 | [12] |
| 3.9 | 62 | 20 | C/10 | 100 | 1,322 | 5.15 | [13] |
| 3.9 | 62 | 20 | C/2 | 200 | 980 | 3.82 | [13] |
| 3.2 | 73.2 | n/a | C/10 | 500 | 1,540 | 4.93 | [14] |
| 2.46 | 50 | 16 | C/2 | 100 | 1,343 | 3.30 | [15] |
| 2 | 69.6 | n/a | C/5 | 400 | 997 | 1.99 | [16] |

| | | | | | | | |
|---|---|---|---|---|---|---|---|
| 2.4 | 70 | n/s | 3C | 1000 | 641 | 1.51 | [17] |
| 1 | 58 | n/a | C/2 | 200 | 1,040 | 1.04 | [18] |
| 1.2 | 72 | 12 | C/2 | 250 | 906 | 1.09 | [19] |
| 2 | 72 | 12 | C/2 | 250 | 760 | 1.52 | [19] |
| 4 | 72 | 12 | C/2 | 500 | 616 | 2.46 | [19] |
| 1.5 | 55.5 | n/a | C/5 | 600 | 912 | 1.37 | [20] |
| 4 | 80 | 10 | C/10 | 100 | 1,036 | 4.14 | This work |
| 4 | 80 | 10 | C/10 | 100 | 1,084 | 4.34 | This work |
| 4 | 80 | 10 | C/10 | 100 | 1,078 | 4.31 | This work |
| 4 | 80 | 10 | C/10 | 100 | 831 | 3.33 | This work |

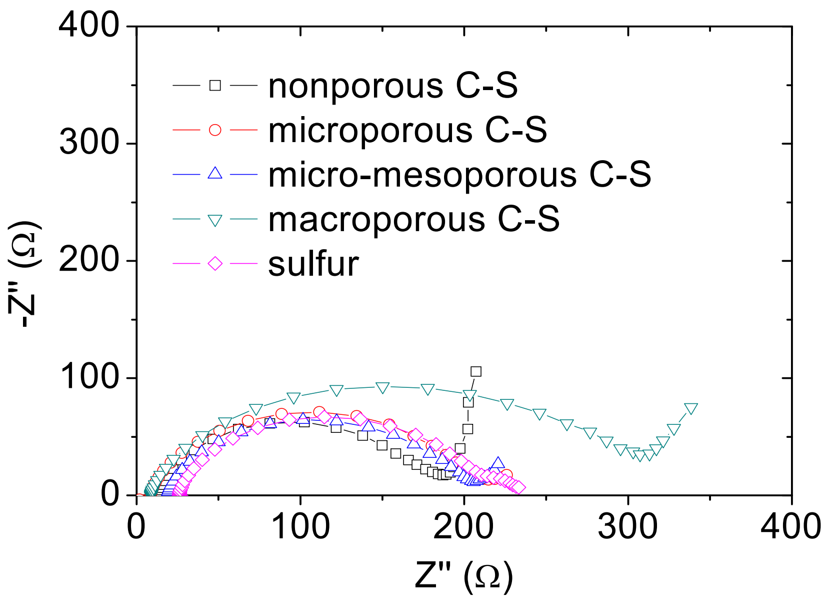


**Figure S1.** Electrochemical impedance analysis of C–S nanocomposites.

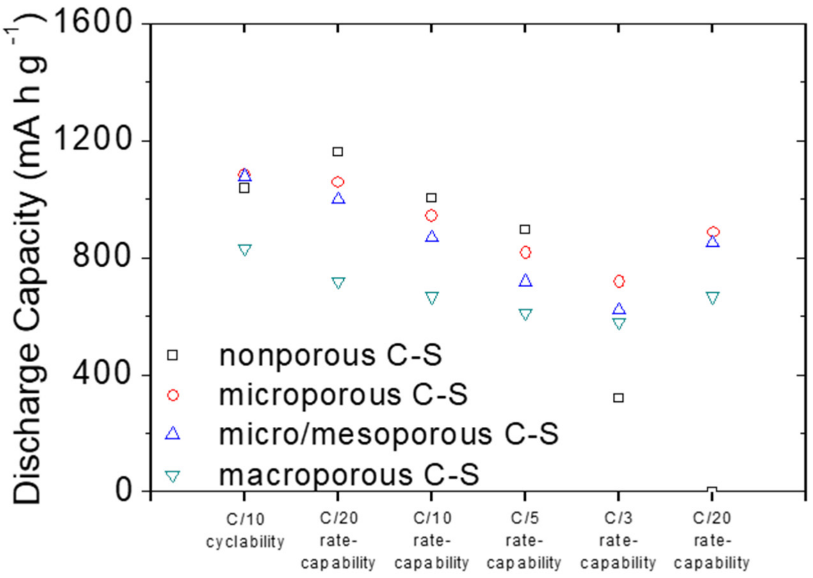


**Figure S2.** The effect of nanoporosity of C–S nanocomposites toward their corresponding electrochemical performance (*i.e.*, cyclability at the C/10 rate with the initial discharge capacity, and the rate capability at C/20–C/3 rates with the capacity at the tenth cycle in each rate).

## References


1. Liu, S.; Li, J.; Yan, X.; Su, Q.; Lu, Y.; Qiu, J.; Wang, Z.; Lin, X.; Huang, J.; Liu, R.; Zheng, B.; Chen, L.; Fu, R.; Wu, D. Superhierarchical cobalt-embedded nitrogen-doped porous carbon nanosheets as two-in-one hosts for high-performance lithium–sulfur batteries. *Adv. Mater.* **2018**, *30*, 1706895, doi:10.1002/adma.201706895.
2. Li, B. Q.; Zhang, S. Y.; Kong, L.; Peng, H. J.; Zhang, Q. Porphyrin organic framework hollow spheres and their applications in lithium–sulfur batteries. *Adv. Mater.* **2018**, 30, 1707483, doi:10.1002/adma.201707483.
3. Chen, K.; Sun, Z.; Fang, R.; Shi, Y.; Cheng, H. M.; Li, F. Metal–organic frameworks (MOFs)-Derived nitrogen-doped porous carbon anchored on graphene with multifunctional effects for lithium–sulfur batteries. *Adv. Funct. Mater.* **2018**, *28*, 1707592, doi:10.1002/adfm.201707592.
4. Yu, Q.; Lu, Y.; Luo, R.; Liu, X.; Huo, K.; Kim, J. K.; He, J.; Luo, Y. In Situ Formation of Copper-Based Hosts Embedded within 3D N-Doped Hierarchically Porous Carbon Networks for Ultralong Cycle Lithium–Sulfur Batteries. *Adv. Funct. Mater.* **2018**, *28*, 1804520, doi:10.1002/adfm.201804520.
5. Chang, Z.; He, Y.; Deng, H.; Li, X.; Wu, S.; Qiao, Y.; Wang, P.; Zhou, H. A Multifunctional Silly-Putty Nanocomposite Spontaneously Repairs Cathode Composite for Advanced Li–S Batteries. *Adv. Funct. Mater.* **2018**, *28*, 1804777, doi:10.1002/adfm.201804777.
6. Chen, W.; Lei, T.; Qian, T.; Lv, W.; He, W.; Wu, C.; Liu, X.; Liu, J.; Chen. B.; Yan, C.; Xiong, J. A new hydrophilic binder enabling strongly anchoring polysulfides for high-performance sulfur electrodes in lithium-sulfur battery. *Adv. Energy Mater.* **2018**, *8*, 1702889, doi:10.1002/aenm.201702889.
7. Yan, M.; Chen, H.; Yu, Y.; Zhao, H.; Li, C. F.; Hu, Z. Y.; Wu, P.; Chen, L.; Wang, H.; Peng, D.; Gao, H.; Hasan, T.; Li. Y.; Su, B. L. 3D Ferroconcrete-Like Aminated Carbon Nanotubes Network Anchoring Sulfur for Advanced Lithium–Sulfur Battery. *Adv. Energy Mater.* **2018**, 8, 1801066, doi:10.1002/aenm.201801066.
8. Guo, Z.; Nie, H.; Yang, Z.; Hua, W.; Ruan, C.; Chan, D.; Ge, M.; Chen, X.; Huang, S. 3D CNTs/Graphene-S-$Al_3Ni_2$ Cathodes for High-Sulfur-Loading and Long-Life Lithium–Sulfur Batteries. *Adv. Sci.* **2018**, *5*, 1800026, doi:10.1002/advs.201800026.
9. Liu, Z.; Zhou, L.; Ge, Q.; Chen, R.; Ni, M.; Utetiwabo, W.; Zhang, X.; Yang, W. Atomic iron catalysis of polysulfide conversion in lithium–sulfur batteries. *ACS Appl. Mater. Interfaces* **2018**, *10*, 19311−19317, doi:10.1021/acsami.8b03830.
10. Gómez-Urbano, J. L.; Gómez-Cámer, J. L.; Botas, C.; Díez, N.; del Amo, J. L.; Rodríguez-Martinez, L. M.; Carriazo, D.; Rojo, T. Hydrothermally reduced graphene oxide for the effective wrapping of sulfur particles showing long term stability as electrodes for Li-S batteries. *Carbon* **2018**, *139*, 226-233, doi:10.1016/j.carbon.2018.06.053.
11. Gueon, D.; Hwang, J. T.; Yang, S. B.; Cho, E.; Sohn, K.; Yang, D. K.; Moon, J. H. Spherical macroporous carbon nanotube particles with ultrahigh sulfur loading for lithium–sulfur battery cathodes. *ACS nano* **2018**, *12*, 226-233, doi:10.1021/acsnano.7b05869.
12. Wang, S.; Hou, X.; Zhong, Z.; Shen, K.; Zhang, G.; Yao, L.; Chen, F. $Co_3O_4$-NP embedded mesoporous carbon rod with enhanced electrocatalytic conversion in lithium-sulfur battery. *Sci. Rep.* **2018**, *8*, 1-9, doi:10.1038/s41598-018-34195-z.

13. Zhang, Y. Z.; Zhang, Z.; Liu, S.; Li, G. R.; Gao, X. P. Free-standing porous carbon nanofiber/carbon nanotube film as sulfur immobilizer with high areal capacity for lithium–sulfur battery. *ACS Appl. Mater. Interfaces* **2018**, *10*, 8749-8757, doi:10.1021/acsami.8b00190.
14. Liu, J.; Zhang, W.; Chen, Y.; Zhou, P.; Zhang, K. A novel biomimetic dandelion structure-inspired carbon nanotube coating with sulfur as a lithium–sulfur battery cathode. *Nanotechnology* **2019**, *30*, 155401, doi:10.1088/1361-6528/aafe46.
15. Shi, H.; Zhao, X.; Wu, Z. S.; Dong, Y.; Lu, P.; Chen, J.; Ren, W.; Cheng, H.-M.; Bao, X. Free-standing integrated cathode derived from 3D graphene/carbon nanotube aerogels serving as binder-free sulfur host and interlayer for ultrahigh volumetric-energy-density lithium–sulfur batteries. *Nano Energy* **2019**, *60*, 743-751, doi:10.1016/j.nanoen.2019.04.006.
16. Kim, J.; Kang, Y.; Song, S. W.; Suk, J. Freestanding sulfur-graphene oxide/carbon composite paper as a stable cathode for high performance lithium-sulfur batteries. *Electrochim. Acta* **2019**, *299*, 27-33, doi:Freestanding sulfur-graphene oxide/carbon composite paper as a stable cathode for high performance lithium-sulfur batteries.
17. Ren, M.; Lu, X.; Chai, Y.; Zhou, X.; Ren, J.; Zheng, Q.; Lin, D. A three-dimensional conductive cross-linked all-carbon network hybrid as a sulfur host for high performance lithium-sulfur batteries. *J. Colloid Interface Sci.* **2019**, *552*, 91−100, doi:10.1016/j.jcis.2019.05.042.
18. Tiwari, V. K.; Song, H.; Oh, Y.; Jeong, Y. Synthesis of sulfur-co-polymer/porous long carbon nanotubes composite cathode by chemical and physical binding for high performance lithium-sulfur batteries. *Energy* **2020**, *195*, 117034. https://doi.org/10.1016/j.energy.2020.117034.
19. Yan, R.; Oschatz, M.; Wu, F. Towards stable lithium-sulfur battery cathodes by combining physical and chemical confinement of polysulfides in core-shell structured nitrogen-doped carbons. *Carbon* **2020**, *161*, 162−168, doi:10.1016/j.carbon.2020.01.046.
20. Xu, H.; Hao, J.; Chen, Y.; You, H.; Liu, X.; Yang, H. High Performance of Sulfur/Carbon Cathode Synthesized via a Facile Green Microwave Approach. *Energy Fuels* **2021**, *35*, 2750-2757, doi:10.1021/acs.energyfuels.0c03699.
